# Evaluation of regression methods when immunological measurements are constrained by detection limits

**DOI:** 10.1186/1471-2172-9-59

**Published:** 2008-10-17

**Authors:** Hae-Won Uh, Franca C Hartgers, Maria Yazdanbakhsh, Jeanine J Houwing-Duistermaat

**Affiliations:** 1 Department of Medical Statistics and Bioinformatics, Leiden University Medical Center, Leiden, the Netherlands; 2 Department of Parasitology, Leiden University Medical Center, Leiden, the Netherlands

## Abstract

**Background:**

The statistical analysis of immunological data may be complicated because precise quantitative levels cannot always be determined. Values below a given detection limit may not be observed (*nondetects*), and data with nondetects are called left-censored. Since nondetects cannot be considered as missing at random, a statistician faced with data containing these nondetects must decide how to combine nondetects with *detects*. Till now, the common practice is to impute each nondetect with a single value such as a half of the detection limit, and to conduct ordinary regression analysis. The first aim of this paper is to give an overview of methods to analyze, and to provide new methods handling censored data other than an (ordinary) linear regression. The second aim is to compare these methods by simulation studies based on real data.

**Results:**

We compared six new and existing methods: deletion of nondetects, single substitution, extrapolation by regression on order statistics, multiple imputation using maximum likelihood estimation, tobit regression, and logistic regression. The deletion and extrapolation by regression on order statistics methods gave biased parameter estimates. The single substitution method underestimated variances, and logistic regression suffered loss of power. Based on simulation studies, we found that tobit regression performed well when the proportion of nondetects was less than 30%, and that taken together the multiple imputation method performed best.

**Conclusion:**

Based on simulation studies, the newly developed multiple imputation method performed consistently well under different scenarios of various proportion of nondetects, sample sizes and even in the presence of heteroscedastic errors.

## Background

The number of immunological parameters that can be measured in large scale epidemiological studies has been rapidly increasing. Not all of these quantitative levels can be determined precisely. Reasons for this lack of precision are that the signal produced by the stimulant is too small for the instrumentation to discriminate the signal from the background noise, or a signal is registered, but certain (laboratory) criteria that identify the substance are not met. Values that cannot be quantified are called *nondetects *(NDs). We assume that all NDs are below a given *detection limit *(DL), and therefore we are dealing with censored data. Simple solutions such as deletion of NDs and single value substitution are often used, but it is unknown to what extent these methods provide unbiased results and thus would be adequate for the analysis. Applying various approaches yielded different parameter estimates in the environmental studies [1,2].

When the number of NDs is rather small, one approach of dealing with NDs is simply dropping NDs and apply linear regression to the remaining data. A second commonly used approach is to substitute NDs with a certain value smaller than the DL (0, DL/2 or DL) and to use linear regression [3,4]. The validity of these approaches will depend on the number and the unknown range of NDs. A third common practice is to dichotomize the cytokine measurements based on a certain cut-off point (DL or median) and to apply logistic regression to this binary variable [3,5]. A major drawback of this approach is that by dichotomizing much information is lost. Note also that the choice of 0, DL/2 or DL in the single value substitution and the threshold in the logistic regression approach is arbitrary. An important issue is then how to decide which method is optimal for a particular data set. Moreover, more sophisticated statistical methods may be needed for analyzing this type of data.

This paper is motivated by a study on the relationship between intensity of parasite infection and cytokines measurements resulting from whole blood assay after stimulation with lipopolysaccharide (Table 1). One of the cytokine measurements has only a small proportion of NDs (5.5%), whereas the second measurement has a relatively large proportion of NDs (66%). In addition to the presence of censored measurements, the distribution of cytokine measurements is often positively skewed. Skewed distributions in biology often closely fit the log-normal distribution and this characterization can be advantageous in the biological system when many factors act in multiplicative ways [6,7]. Therefore, it can be assumed that the cytokine variables are normally distributed after an appropriate (log-)transformation.

**Table 1:** Description of cytokine data

Cytokine	sample size	proportion of NDs	DL
1	181	5.5%	10 pg/ml
2	173	66%	5 pg/ml

Considering the efforts made by collecting data, it seems worth while to investigate sophisticated and (maybe) time-consuming statistical methods to analyze data appropriately [8]. In this paper we review several commonly used methods in immunology and more advanced methods used in other fields such as environmetrics and econometrics [1,9,10]. A second goal is to evaluate the performances of these methods via simulation studies [2,11,12]. The validity and precision of simple methods such as deletion and single value substitution will be studied for various scenarios including different proportions on ND's and different error models. In addition the utility of advanced statistical methods will be quantified.

## Results and discussion

### Results of simulation studies

In Table 2, the six methods for analyzing data containing NDs that were considered are summarized: removal of nondetects (DELETION), single substitution of NDs with half of the value of DL (DL/2), extrapolation by regression on order statistics (ROS), multiple imputation using maximum likelihood estimation (MI), tobit regression (TOBIT), and logistic regression (LOGIT). To study the performance of these methods, we simulated data sets of size 200, 400 and 1,000 with proportions of NDs of 10, 30, 50 and 70%.

**Table 2:** Methods used for comparing

Methods	Description	Software	Disadvantage
Deletion	Remove NDs.	Any statistical package	Bias
DL/2	Substitute each ND with half of the value of DL.	Any statistical package	Large RMSE for large proportion of NDs
ROS	After computing a linear regression for data versus their normalized scores below-DL values are extrapolated under distributional assumption.	R-package NADA [24,27]	Underestimation of variance for large proportion of NDs
MI	Estimation of mean and standard deviation by MLE. Creating 10 complete samples. Pool the results from 10 individual analyses.	R (software available on request)	Bias for small proportion of NDs
TOBIT	Parametric estimation method for incorporating NDs.	R, Stata [28], SAS [29]	Sensitive to heteroscedastic errors
LOGIT	Create binary dependent variable of NDs (0s) and detects (1s).	Any statistical package	Loss of information & parameter estimates are less interpretable

**Figure 1:**
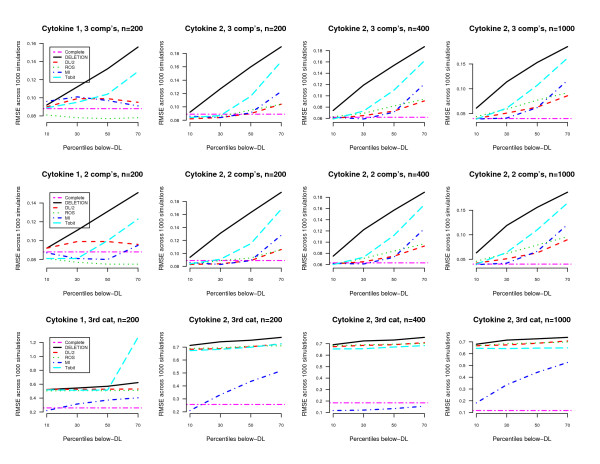
**Simulation results regarding RMSE from different settings**. The five methods were compaired: removal of nondetects (DELETION), single substitution of NDs with half of the value of DL (DL/2), extrapolation by regression on order statistics (ROS), multiple imputation using maximum likelihood estimation (MI), tobit regression (TOBIT).

Regarding RMSE we summarized results in Figure 1. The considered simulation settings were as follows. The plots on the first column represent the scenario with negative effect imitating Cytokine 1, whilst the second to fourth columns show the positive effect of malaria intensity (Cytokine 2). The Columns also show the effect of different thresholds. For the plots of the first column the cut-off points of DL values were relatively large with 14.7, 10, 17, and 29 pg/ml corresponding to 10, 30, 50 and 70% of the whole data sets. In contrast, the second to fourth columns the DL-values were very close to zero, namely 0.7, 1.6, 2.7, and 4.6 pg/ml.

The three rows display the results from the three different covariates. For the quantitative covariates generated from three-component mixture (row 1) and two-component mixture (row 2), the simulation results were similar. Therefore, the results from the three-component mixture imitating Cytokine 1 are discussed in details.

**Figure 2:**
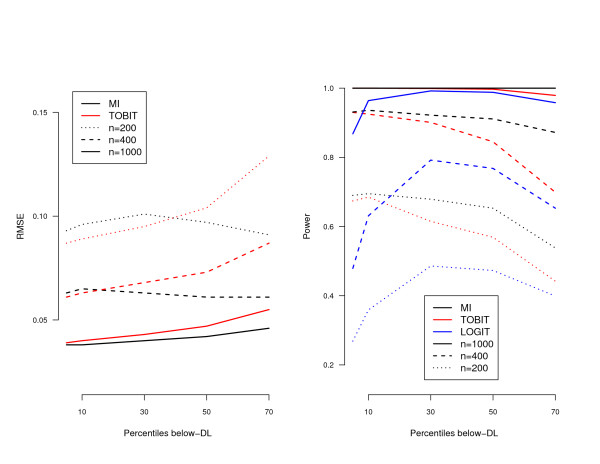
**Comparison of MI, TOBIT and LOGIT methods**. RMSE and power averaged across 1000 simulated data sets of sample size 200, 400 and 1,000. The *black, red *and *blue *colors indicate MI, TOBIT and LOGIT methods, respectively.

In Additional file 1 the results were summarized in terms of bias, root mean square error (RMSE) and coverage probability of 95% confidence interval (CI). Entries in the table are averages of 1,000 replications. In terms of bias, at all levels of NDs and for all sample sizes, the Deletion method produced the least accurate estimates. In contrast, for small data sets containing a small proportion of NDs (size 200 and 10% NDs), the TOBIT model produced nearly unbiased estimates, while the DL/2 method also performed well. With respect to RMSE, the MI method performed best in general. Note that the ROS method produced the smallest RMSE values, although it produced relatively large biases. This indicates that ROS underestimates the variance. For visualization of the results in terms of RMSE, the performance of the two best-performing methods – TOBIT and MI – were compared for different sample sizes in Figure 2. The advantage of using the TOBIT model with small percentage of NDs seems to disappear with increasing sample sizes.

In Table 3 the averaged variances of parameter estimates were given for the TOBIT, MI, and LOGIT methods. The variances of the TOBIT model increased with increasing proportion of NDs, while the level of variance using the MI method remained stable throughout the different sample sizes and the percentages of NDs. The LOGIT method with the proportion of NDs of 30% and 50% produced smaller variances than with a very small and very large proportions of NDs.

**Table 3:** Variance of estimates provided by the MI, TOBIT and LOGIT approaches at various proportions of nondetects (entries are averages of 1000 repetitions)

	Sample size of 200			Sample size of 400			Sample size of 1000		
% NDs	MI	TOBIT	Logit	MI	TOBIT	Logit	MI	TOBIT	Logit
10%	0.0050	0.0044	0.0358	0.0022	0.0022	0.0164	0.0008	0.0009	0.0063
30%	0.0055	0.0050	0.0185	0.0022	0.0025	0.0089	0.0007	0.0010	0.0035
50%	0.0055	0.0061	0.0195	0.0021	0.0030	0.0095	0.0006	0.0012	0.0037
70%	0.0048	0.0092	0.0319	0.0018	0.0043	0.0147	0.0005	0.0017	0.0056

Regarding efficiency of the methods, the right panel of Figure 2 shows the power to detect at the nominal significance level of *α *= 5% for the TOBIT, MI, and LOGIT methods. The MI method was the best at all proportions of NDs and for all sample sizes. For a small proportion of NDs, the performance of the TOBIT and MI methods was equivalent. Overall, the LOGIT method performed worst.

**Figure 3:**
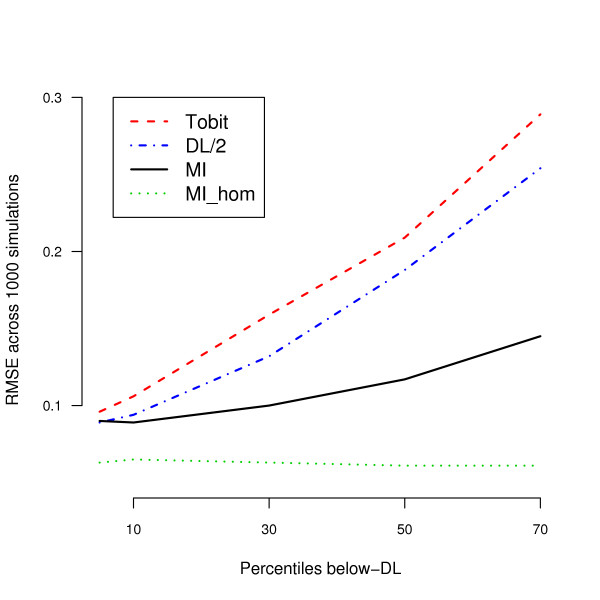
**Comparison of MI, TOBIT, and DL/2 methods with heteroscedastic errors**. Results of RMSE across 1000 simulated data sets of sample size 200, 400 and 1,000. The green dotted line indicates the Mi method based on homoscedastic errors.

Additionally we compared the performance of TOBIT, DL/2 and MI approaches under heteroscedastic errors. The results are depicted in Figure 3. The RMSE of the TOBIT and DL/2 methods increased rapidly with increasing proportion of NDs. The MI approach appeared to be most robust and RMSE was below 0.1 for proportions of ND under 30%.

In the last row of Figure 1, the results for the third (microscopic) category with reference to negative category are given. In contrast to quantitative covariates, there were dissimilarities in the behavior. RMSE did not increase as rapidly as with quantitative covariates, when the proportion of NDs becomes large. However, the actual RMSE values were much higher. Although the order of the best methods did not vary much, the TOBIT model gave bad performance when the sample size was small (n = 200).

### Application to immunological data

**Figure 4:**
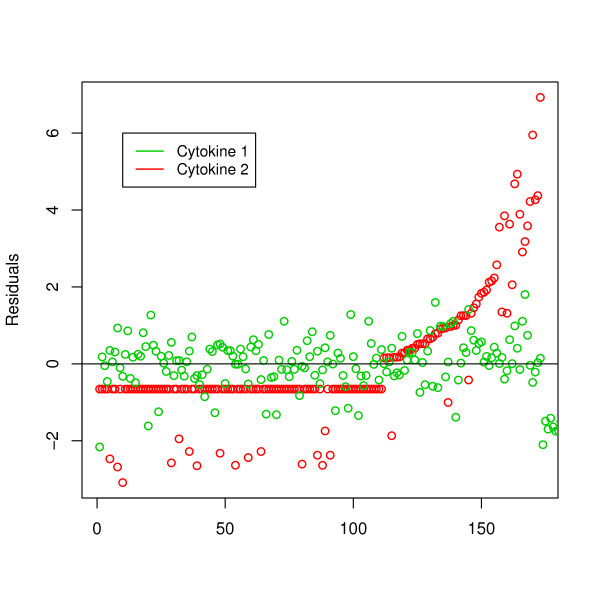
**Residuals**. Residuals by conducting linear regression with a single substitution of NDs. The green and red colors represent the residuals of Cytokine 1 and Cytokine 2, respectively.

Different choices yielded different results as illustrated for our motivating data as can be seen in Table 4. To determine the optimal method for this particular data set, simulation results are used as a reference. For the two cytokine responses, the residuals of simple linear regression on intensity of parasite infection using DL/2 imputation for NDs are given in Figure 4.

**Table 4:** Application to real data: for the LOGIT model two cut-off points were used, median for Cytokine 1 and DL for Cytokine 2

	Methods	$\hat{\beta }$ (Slope)	SE($\hat{\beta }$)	$\hat{\beta }$ SE($\hat{\beta }$)	*p*-value
**Cytokine 1**	**Standard methods**				
					
	Deletion	0.012	0.042	0.277	0.782
	Substitution of 0	-0.255	0.055	-4.645	< 0.0001
	Substitution of DL/2	-0.186	0.047	-3.951	0.0001
	Substitution of DL	-0.117	0.040	-2.879	0.005
	LOGIT (Median)	-0.198	0.139	-1.423	0.155
					
	**Advanced methods**				
					
	ROS	-0.085	0.039	-2.184	0.030
	TOBIT	-0.190	0.048	-3.960	0.00008
	MI	-0.149	0.046	-3.264	0.0006

**Cytokine 2**	**Standard methods**				
					
	Deletion	0.801	0.129	6.193	< 0.0001
	Substitution of 0	0.585	0.1101	5.311	< 0.0001
	Substitution of DL/2	0.545	0.093	5.842	< 0.0001
	Substitution of DL	0.504	0.079	6.418	< 0.0001
	LOGIT (DL)	0.208	0.113	1.841	0.066
					
	**Advanced methods**				
					
	ROS	0.497	0.081	6.135	< 0.0001
	TOBIT	0.792	0.179	4.436	< 0.0001
	MI	0.550	0.127	4.322	< 0.0001

For Cytokine 1, considering the rather small sample size (181), small proportion of NDs (5.5%), and homoscedastic errors (Figure 4) the TOBIT method (with parameter estimate $\hat{\beta }$ = 0.190 and the corresponding *p*-value = 0.00008) might be a good choice. The simple DL/2 method ($\hat{\beta }$ = -0.186) gave similar results, which confirmed the simulation results. The MI method gave the next best estimate $\hat{\beta }$ = -0.149. Logistic regression using the median value as a cut-off point (*p*-value = 0.155) resulted in loss of power, and the estimate by the ROS method ($\hat{\beta }$ = -0.085) was greatly biased. It was noted that the estimate by the Deletion method ($\hat{\beta }$ = 0.012) was of different direction even with this small proportion of NDs.

Next we consider Cytokine 2 as outcome variable. As can be derived from Figure 4, heteroscedasticity of errors was indicated for Cytokine 2. Based on the simulation results, for a large number of NDs and for heteroscedastic errors the MI method (with parameter estimate $\hat{\beta }$ = 0.550 and the corresponding SE($\hat{\beta }$) = 0.113) might be preferable to others. The DL/2 method ($\hat{\beta }$ = 0.545 and SE($\hat{\beta }$) = 0.093) yielded a similar effect estimate, but the standard error of the parameter estimate was smaller compared to the MI method. Since the proportion of NDs was larger than the median, DL was used as a cut-off point for logistic regression. The use of binary rather than continuous data caused loss of power and the estimate was not even significant any more. The results given by the ROS method were greatly biased. Finally, as could be expected from the simulation study the TOBIT method overestimated the effect size ($\hat{\beta }$ = 0.792).

## Discussion

In the field of immunology there is great need for specialized methods for analysis of data in order to improve accuracy and power. In this paper we proposed advanced methods to deal with data sets when DL plays a significant role. Via simulation studies we first evaluated performances of several methods. Because NDs are not missing at random, biases can be expected when simply dropping NDs. Even with proportion of NDs of 10%, the bias was unacceptable. For parameter estimation substituting DL/2 in NDs was reasonable, but the variance was underestimated. Furthermore, as illustrated by our data set the choice of the imputed value (0, DL/2, DL) remains an issue. For large proportion of NDs, ROS appeared to yield large biases. Analogous to the DL/2 method, the variance of parameter estimates was underestimated. The TOBIT method appeared to be an elegant method to deal with a small proportion of NDs under the constant error assumption. If possible, the normality assumption should be checked before considering the TOBIT model (Figure 4) [13]. For larger proportions of NDs (larger than 10%), MI outperforms the other methods in terms of RMSE. Since imputations are multiple, the MI method takes into account the uncertainty about the true values of the NDs. Furthermore, it is rather robust against heteroscedasticity of errors. Figure 2 showed for large sample size the MI method produces more accurate estimates than the TOBIT method. Note that the MI method might be improved by using more sophisticated methods to compute the mean and the standard deviation of a truncated normal distribution [14]. However, diminishing variances by increasing proportion of NDs require the careful use of the MI method when proportion of NDs is greater than 50%.

We also compared results from the different scenarios: (1) whether there is positive relationship between dependent and independent variables, (2) when the characteristics of covariates were changed (three- and two component mixture, or categorized), and (3)whether the closeness of the detection limit to zero will influence the results (Figure 1). In general, the type of included covariates in the model did not influence the findings. Therefore, our findings in this paper can be used as a reference. Nevertheless, careful consideration should be given to what are the appropriate methods for analyzing each specific data.

The limitation of our simulation study lies in skewed error distributions. However, we studied a simple solution of dichotomizing a continuous variable. Although this is an inefficient approach, and determination of cut-off points remains arbitrary [5], for some situations creating a binary outcome variable could be the most sensible option when measurements can easily be categorized. The method can also be extended to more than two categories by using ordered logistic regression (or proportional odds model). Note that to reflect the natural ordering of the categories, ordered logistic regression should be preferred to multinomial logistic regression [15,16]. Additional advantage of using ordered logistic regression is that the results can be presented in one parameter. In contrast, in using multinomial logistic regression as in our simulation study, the first (or most common) level will be considered as reference category (negative level), and the inference of remaining two categories compared to the reference will be given. Although making more categories than two might improve performance, the determination of categories remains arbitrary.

When data are very skewed and normality cannot be achieved by the usual transformation, quantile regression could be considered [17]. This is an econometric regression model, in which a specified conditional quantile (or percentile) of the outcome variable is expressed as a linear function of covariates.

Simply the lines split the population into two parts with the proportion of 70, 80 or 90% lying below the line, and the proportion 30, 20, or 10% above the line, respectively. Similar to logistic regression the choice of the quantile is arbitrary. However, it assumes no underlying distribution, and is reported to be robust against heteroscedastic errors. Good performance of quantile (or median) regression method have been reported elsewhere [18]. However, when the proportion of NDs is greater than 50%, median regression is not suitable. Also with normally distributed data (after appropriate transformation), the improvement using median regression would be little. The computation of quantile regression is possible using R, SAS, and Stata.

In this paper we considered a single variable restricted with NDs. Extending to multiple regression, such as multiple cytokine measurements of the same individuals and/or related cytokine levels within some set, it is very probable that we encounter NDs in more than one covariate and with different DLs. It can be expected that the large number of correlated cytokine variables would enhance the advantage of using the multiple imputation techniques [19]. In fact, using information on other correlated variables such as families would improve the performance of MI. It is not the purpose of this paper to stress that the MI method should be used everywhere in the presence of DL. Nevertheless, we showed that the search for new methods might gain deeper understanding of data, and that simulation studies can contribute to decide the optimal methods for measurement data with NDs.

## Conclusion

We showed that a dichotomization of continuous variable generally causes loss of information, hence loss of power. We compared the several linear regression methods to deal with the data containing NDs based on simulation studies. The TOBIT method produced the most accurate estimates with the least bias. When the amount of NDs is relatively small (≤ 30%) and the normality assumption is met as Cytokine 1 in our example data, the use of the TOBIT method is recommended. However, as reported elsewhere [20,21], the TOBIT model is sensitive to the violation of normality assumption. Therefore, when heteroscedastic errors are suspected, and/or the amount of NDs is large, robust statistical methods have to be considered. We proposed to employ multiple imputation technique. The MI method performed consistently well under different scenarios of various proportion of NDs (≤ 50%), sample sizes and even in the presence of heteroscedastic errors.

## Methods

### Methods to compare

The following linear regression model is considered for the outcome *y*_*i *_of subject *i *with a covariate *x*_*i*_

(1) *y*_*i *_= *α *+ *βx*_*i *_+ *ε*_*i*_,

where *ε*_*i *_is random noise and *i *= 1, ..., *n*. The error *ε *is assumed to be uncorrelated with *x *and to have a mean equal to zero and a constant variance. The parameters *α *and *β *denote the intercept and the average change in *y *with *x*. By Ordinary Least Squares (OLS) the estimated slope and intercept of the regression line can be computed. However, in immunological data the *y*s in equation (1) are only partly observed. A lower threshold or detection limit, DL, interferes with measurements of low levels as follows:

(2) $$\begin{array}{lllll} y_{i}^{*} & = & y_{i} & \text{if} & y_{i}^{*}>\text{DL}\\ y_{i}^{*} & = & \text{NDs}, & \text{if} & y_{i}^{*}\leq \text{DL}. \end{array}$$

Since NDs of cytokine measurements reflect levels of exposure, they cannot be considered as *missing at random *(MAR) [22]. Therefore, deleting the lowest values is expected to produce biased results. Other types of methods to analyze these data are imputation and modelling of NDs. An overview of the available methods is given in Table 1. In environmental statistics a method called robust regression on order statistics (ROS) approach exists [1,9]. This method is often used to compute summary statistics.

To reflect uncertainty about imputation, we propose to employ multiple imputation approach as introduced by Little and Rubin [22,23]. Based on a truncated normal distribution, we first compute the mean and the standard deviation. This can be done using the functions cenmle or ros from the R-package NADA [24]. Then, the values for NDs were generated randomly and *m *complete data sets are created and each data set is analyzed separately. Rubin (Chapter 3, [25]) gives the following rule for combining the results. With *m *imputations, we obtain *m *different sets of the point estimate ${\hat{\beta }}_{i}$ as well as standard errors *s*_1_, ..., *s*_*m*_. The pooled MI point estimate is then simply the average of the *m *estimates: $\bar{\beta }=\frac{1}{m}\sum_{j=1}^{m}{\hat{\beta }}_{i}$.

The variance estimate associated with $\bar{\beta }$ has two components. The *within-imputation variance *can be estimated by the average of the complete data variance $\bar{U}=\frac{1}{m}\sum_{j=1}^{m}s_{i}^{2}$. The *between-imputation variancem *is the variance of the estimate $\bar{\beta }$, $B=\frac{1}{m-1}\sum_{j=1}^{m}{\left({\hat{\beta }}_{1}-\bar{\beta }\right)}^{2}$ The *total variance*is defined by *T *= *Ū *+ (1 + *m*^-1^)*B *and inferences are based on the approximation $\bar{\beta }$/*T*^-1/2 ^~ *t*_*ν*_, where the degrees of freedom are given by $\nu =(m-1)\left[1+\frac{\bar{U}}{(1+m^{-1})B}\right]$.

Finally, two non-imputation methods for incorporating NDs into regression models are investigated. Without adding uncertainty on the distribution of the NDs, the outcomes can be dichotomized and logistic regression can be applied. However, the relationship between the covariate and the outcome is now on a logit scale instead of a linear one. A more sophisticated approach is to use maximum likelihood estimation (MLE) method for left-censored data, called TOBIT model after the economist James Tobin [26]. The model is written as a combination of

$$\begin{array}{cccccc} y_{i}^{*} & = & y_{i} & \text{if} & y_{i}>\text{DL} & \left(\text{OLS part}\right)\\ y_{i}^{*} & = & \text{DL} & & \text{otherwise} & (\text{probit part}). \end{array}$$

The probit part determines whether the outcome variable is below-DL, and the OLS part is a truncated regression model. The TOBIT model estimates a regression model for the data above DL, and assumes that the censored data (below DL) have the same distribution of errors as the observed data. The weakness of this method is that it may be more vulnerable to violation of the assumptions about the error distribution. Many comments can be found in the literature that in the presence of heteroscedasticity the Tobit estimates are inconsistent, and that there is only limited information about the direction of the bias [20,21].

### Simulation study

We simulated data sets by drawing samples from a population similar to the example data in the Background section, and by allocating a proportion of observations as NDs.

For the covariate *x *(infection intensity) we used (1) a three-component normal mixture distribution, (2) a two-component normal mixture distribution, and (3) three classes. The three-component normal mixture distribution has means equal to 0.77, 3.35 and 4.59 and a within-component variance of 0.027. The proportions of the three components were 0.83, 0.13 and 0.04, respectively. The two-component normal mixture distribution has means equal to 0.77 and 3.69 and a within-component variance of 0.069, with their proportions 0.84 and 0.16, respectively.

Then, based on the characteristic of Cytokine 1, outcome variables were generated using the following regression model,

(3) *y*_*i *_= 3.04 0 - 16*x*_*i *_+ *ε*_*i*_,

for individual *i *∈ {1, ..., *n*}. Based on Cytokine 2, we generated outcome variables as

*y*_*i *_= 0.66 + 0.27*x*_*i *_+ *ε*_*i*_.

And, *ε *were assumed to be standard normally distributed.

Based on biology, the malaria parasite measurements lend to be categorized in three classes: negative, submicroscopic, and microscopic. Instead of looking at the effect of malaria with continuous measurements, we considered the categorical malaria variable, say *z*. The dummy code *z*_*i *_= (*z*_*i*1 _*z*_*i*2 _*z*_*i*3_)^⊤ ^denotes a vector of malaria category indicators for the *i*th subject, with elements *z*_*ij *_= 1 if *i*th subject has *j*th category; otherwise *z*_*ij *_= 0. The categorical covariate vector *z *were then generated following the multinomial distribution of categorized malaria status with proportions of 0.69, 0.14, and 0.17. Based on Cytokine 1, *y *were generated following the model:

(4) *y*_*i *_= 2.97 - 0.13*z*_*i*2 _- 0.58*z*_*i*3 _+ *ε*,

while based on Cytokine 2

(5) *y*_*i *_= 0.84 + 0.13*z*_*i*2 _+ 0.77*z*_*i*3 _+ *ε*.

Here *ε *were assumed to be standard normally distributed.

We then considered data samples of size *n *= 200, 400 and 1, 000. The proportions of NDs were set 10%, 30%, 50% and 70%. The corresponding cut-off points of DL values were: (1) for imitation of Cytokine 1, 14.7, 10, 17, and 29 pg/ml, and (2) for mimicking Cytokine 2, 0.7, 1.6, 2.7, and 4.6 pg/ml.

For studying the effect of heteroscedastic errors we used the same model as in (3) but now with a variance depending on the value of *x *by using *ε *~ *N*(0, $\sqrt{x}$).

### Evaluation of methods

In general, accuracy of estimate can be evaluated by bias, which represents the closeness to the true values, and precision measures the ability to repeat a previous estimates (regardless of accuracy). The combination of both accuracy and precision of estimate can be investigated by the root mean square error (RMSE) as follows:

$$\text{RMSE}=\sqrt{{\text{bias}}^{2}+\text{Variance}}.$$

Therefore, parameter estimates provided by the various methods were compared in terms of mean bias and RMSE. Also coverage probability was provided, which is the probability that the confidence interval of the estimates contains the value. Additionally, for the unbiased methods performances were also compared for their hypothesis testing abilities in terms of power. The Wald-type statistic $\hat{\beta }$/SE($\hat{\beta }$) was used for testing. It is approximately distributed as a *t*-distribution with *n *– 2 degrees of freedom for *n *observations in each sample for continuous outcome.

All computations have been done using the program language R [27].

## Authors' contributions

H-WU performed simulation, analyzed the data, and drafted the manuscript. JH-D participated in interpretation of statistical methods, conception and design of the study. MY and FH participated in discussion on biological issues and provided data. All authors edited and approved the written manuscript.

## Supplementary Material

Additional file 1 **Results of simulation studies in accuracy and precision.** Results were obtained by different approaches at various proportions of nondetects (entries are averages of 1000 repetitions). Click here for file
