# Supplementary material for: Evaluation of regression methods when immunological measurements are constrained by detection limits
Source: BMC Immunol. 2008 Oct 17;9:59. doi: 10.1186/1471-2172-9-59 (PMC2592244; doi:10.1186/1471-2172-9-59)
Supplement: Additional file 1 — Results of simulation studies in accuracy and precision. Results were obtained by different approaches at various proportions of nondetects (entries are averages of 1000 repetitions). [file 1471-2172-9-59-S1.pdf]

		Sample size of 200			Sample size of 400			Sample size of 1000		
% NDs	Methods	Bias	RMSE	Coverage[1]	Bias	RMSE	Coverage	Bias	RMSE	Coverage
**Complete**		0.001	0.088	96.0	0.002	0.061	95.2	0.000	0.039	94.0
10%	Deletion	0.051	0.092	96.6	0.053	0.074	96.5	0.053	0.061	96.2
	DL/2	-0.005	0.090	96.2	-0.005	0.064	95.7	-0.004	0.042	93.3
	ROS	0.015	0.081	96.0	0.018	0.058	96.0	0.019	0.039	95.7
	MI	-0.015	0.096	94.3	-0.005	0.065	94.1	0.007	0.038	95.4
	TOBIT	-0.002	0.089	94.9	0.002	0.063	95.6	0.000	0.040	94.8
30%	Deletion	0.085	0.112	96.1	0.087	0.099	97.0	0.088	0.092	96.5
	DL/2	-0.008	0.099	95.1	-0.009	0.071	95.1	-0.009	0.045	96.3
	ROS	0.033	0.078	97.5	0.031	0.058	96.7	0.033	0.044	96.7
	MI	-0.020	0.101	95.5	0.000	0.063	96.0	0.021	0.040	95.4
	TOBIT	0.001	0.095	94.3	-0.002	0.068	95.0	0.000	0.043	93.8
50%	Deletion	0.106	0.132	96.7	0.105	0.117	97.1	0.108	0.112	96.6
	DL/2	0.004	0.099	97.8	0.000	0.070	97.4	0.002	0.044	97.2
	ROS	0.040	0.077	96.9	0.040	0.060	96.1	0.039	0.047	95.8
	MI	-0.014	0.097	97.2	0.008	0.061	97.2	0.030	0.042	96.2
	TOBIT	-0.007	0.104	95.3	-0.003	0.073	95.7	-0.004	0.047	95.8
70%	Deletion	0.122	0.156	96.5	0.121	0.136	96.7	0.120	0.125	96.8
	DL/2	0.035	0.095	97.9	0.038	0.072	98.0	0.038	0.053	98.4
	ROS	0.049	0.078	96.9	0.047	0.063	96.2	0.047	0.053	96.7
	MI	0.018	0.091	97.5	0.027	0.061	96.2	0.038	0.046	95.5
	TOBIT	-0.011	0.129	93.9	-0.002	0.087	95.9	-0.004	0.055	94.9

[1] probability of 95% CI
